# Stress and polyamine metabolism in fungi

**DOI:** 10.3389/fchem.2013.00042

**Published:** 2014-01-10

**Authors:** Laura Valdés-Santiago, José Ruiz-Herrera

**Affiliations:** Departamento de Ingeniería Genética, Unidad Irapuato, Centro de Investigación y de Estudios Avanzados del Instituto Politécnico NacionalIrapuato, México

**Keywords:** polyamines, stress response, polyamine mutants, fungi, metabolism

## Abstract

Fungi, as well as the rest of living organisms must deal with environmental challenges such as stressful stimuli. Fungi are excellent models to study the general mechanisms of the response to stress, because of their simple, but conserved, signal-transduction and metabolic pathways that are often equivalent to those present in other eukaryotic systems. A factor that has been demonstrated to be involved in these responses is polyamine metabolism, essentially of the three most common polyamines: putrescine, spermidine and spermine. The gathered evidences on this subject suggest that polyamines are able to control cellular signal transduction, as well as to modulate protein-protein interactions. In the present review, we will address the recent advances on the study of fungal metabolism of polyamines, ranging from mutant characterization to potential mechanism of action during different kinds of stress in selected fungal models.

## Introduction

Sudden changes in the external conditions can directly impact the internal environment of all living organisms, and can disrupt their homeostasis and normal physiology. Therefore, cells have developed complex systems to identify the status of their environment, and rapidly generate defense systems against environmental stress (Gasch, 2007; Lushchak, 2011; Montibus et al., 2013). An effective procedure to obtain information on the mechanisms regulating the stress response is the identification of pathways or specific genes suffering changes in their expression under stress conditions. Accordingly, it has been shown that a large number of genes are affected in their expression when an organism responds to an environmental stress. Many of these genes are conserved among fungi, including those involved in carbohydrate metabolism, protein metabolism, defense against reactive oxygen species (ROS), intracellular signaling, etc. (Giaever et al., 2002; Gasch, 2007). Among the known elements related with stress response are polyamines, as it has been widely demonstrated in plants (Galston and Sawhney, 1990; Alcazar et al., 2010; Gill and Tuteja, 2010a; Gupta et al., 2013). However, in recent years, investigations into the molecular genetics of fungal polyamine metabolism have led to the isolation of mutants altered in this function that show alterations in their response to stress. The importance of polyamine participation in the response to stress has been highlighted by the increasing number of reports describing the changes occurring in polyamine concentrations induced in response to diverse forms of stress. Accordingly, in the present review we describe the recent genetic and molecular evidences illustrating the role of polyamine metabolism in the responses of fungi to stress.

## Polyamine metabolism in fungi

### Biosynthesis of polyamines

**Figure 1:**
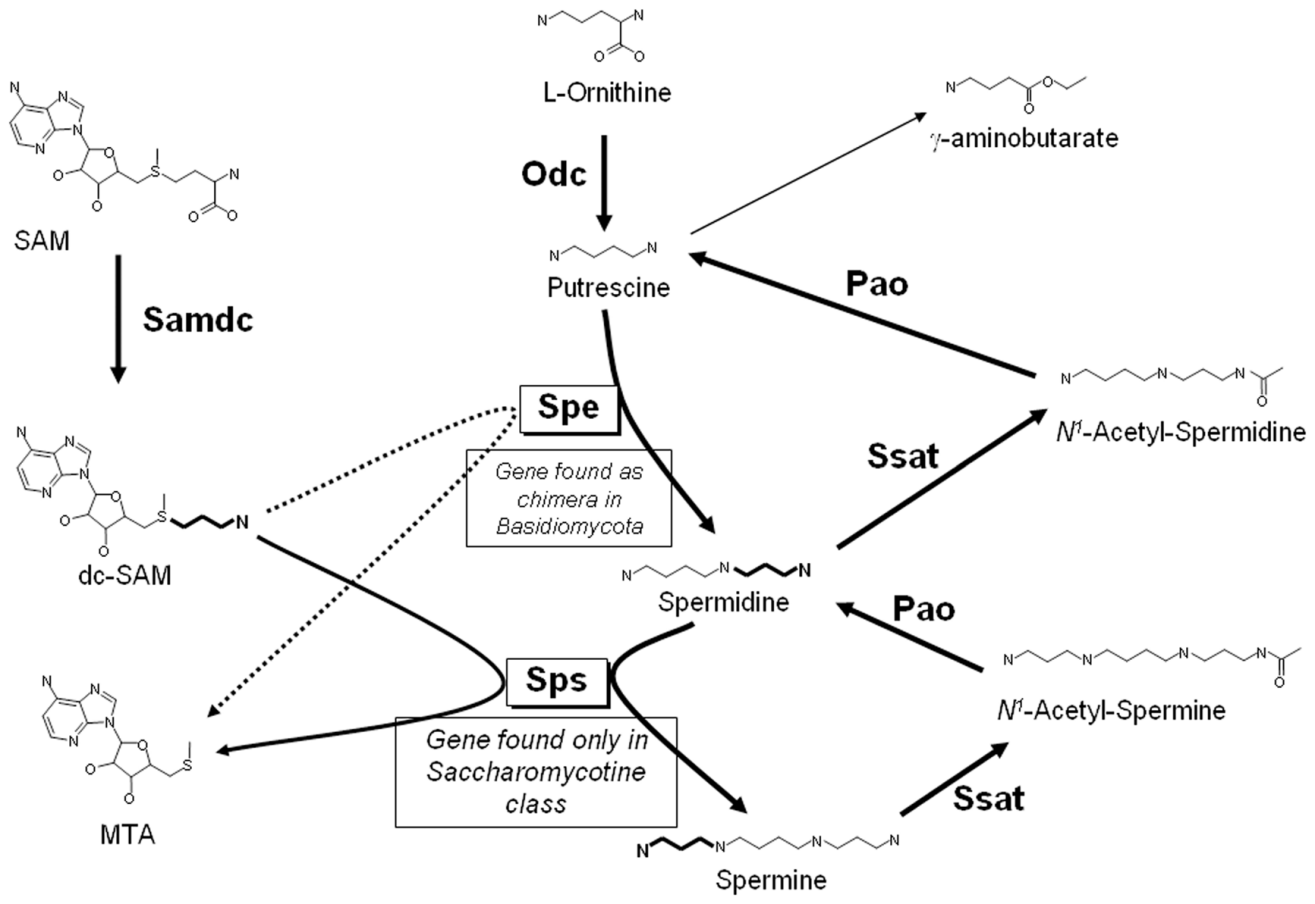
**General pathway for the biosynthesis and catabolism of polyamines in fungi**.

Putrescine, spermidine and spermine are low-molecular-mass aliphatic cations critical to cell survival (Tabor and Tabor, 1984), and the pathways for their biosynthesis have been analyzed in all the kingdoms of living organisms [e.g., see Valdes-Santiago et al., 2012a]. However, there are some basic differences in the distribution of polyamines among them. In general, it is accepted that in fungi, as well as in animals, there is only one mechanism to produce putrescine *de novo*: by decarboxylation of ornithine by the enzyme ornithine decarboxylase (Odc, E.C.4.1.1.17), which is the first and rate-limiting enzyme in the synthesis of polyamines. In most fungi decarboxylation of ornithine is the only route to putrescine synthesis, however, in plants there is an additional route to produce putrescine: the decarboxylation of L-arginine by arginine decarboxylase (for more details of the general pathway in plants refer Alcazar et al., 2010; Gupta et al., 2013). Nevertheless, some authors have reported arginine decarboxylase activity in fungi such as *Ceratocystis minor, Verticillium dahliae, Colletotrichum gleosporoides*, and *Gigaspora rosea*, suggesting that both, arginine and ornithine decarboxylase enzymes could be involved in putrescine biosynthesis (Khan and Minocha, 1989; Weerasooriya et al., 2003; Sannazzaro et al., 2004). Putrescine is converted to spermidine by the addition of an aminopropyl group. *S*-adenosylmethionine decarboxylase (Samdc; E.C.4.1.1.50) is responsible for the formation of the donor of the aminopropyl group, decarboxylated S-adenosylmethionine (dcSAM), and spermidine synthase (Spe, E.C.2.5.1.16) is the transferase of the aminopropyl group from dcSAM to putrescine. Finally, spermidine is converted to spermine by a similar reaction, resulting in the transfer of an aminopropyl group to spermidine by spermine synthase (Sps; E.C.2.5.1.22) (see Figure 1). It should be noticed that this last step does not occur in most fungi, which accordingly contain only putrescine and spermidine (Nickerson et al., 1977). An ortholog of the gene encoding Sps is found only in the subphylum Saccharomycotina of the Ascomycota phylum which include few human pathogens and at least 10 phytopathogenic species (Suh et al., 2006; Pegg and Michael, 2010). Also interesting is to mention that *SPE* gene is present in members of Basidiomycota subphyla as a bifunctional gene encoding spermidine synthase and saccharopine dehydrogenase, the last enzyme involved in lysine biosynthesis (Leon-Ramirez et al., 2010). Regarding polyamine distribution, in general, eukaryotes have low putrescine content and a high content of spermidine and spermine, while prokaryotes have a higher concentration of putrescine than spermidine (Manni et al., 1987). A difference between fungi and plants is the presence of thermospermine, an isomer of spermine that has not been found in fungi (Fuell et al., 2010; Takano et al., 2012)

### Retro-conversion of polyamines

In fungi, polyamines are oxidized to putrescine by the pathway shown in Figure 1. The first step is the acetylation of the aminopropyl group of polyamines, a reaction catalyzed by spermine or spermidine *N*^1^—acetyltrasferase (Ssat; E.C. 2.3.1.57), to give either *N*^1^—acetyl-spermidine or *N*^1^—acetyl-spermine. These are in turn degraded by a polyamine oxidase (Pao; E.C. 1.5.3.11), with the formation of either putrescine or spermidine. Polyamine acetyltransferases and polyamine oxidases have been reported to be present in yeast as well as in other fungi (Yamada et al., 1980; Chattopadhyay et al., 2003b; Landry and Sternglanz, 2003; Liu et al., 2005; Valdes-Santiago et al., 2010). The ability to direct back-conversion of spermine to spermidine by spermine oxidase such as occurs in animals has been reported in plants and it is other dissimilarity between plants and fungi (Tavladoraki et al., 1998; Alcazar et al., 2006). In plants, redundancy of genes such as diamino oxidases or polyamine oxidases genes has been documented (Alcazar et al., 2006; Ono et al., 2012).

### Regulation of polyamine synthesis

Modulation of polyamine biosynthesis is mostly achieved by the degradation of Odc protein, as it has been reported in different species including *Schizosaccharomyces pombe, Neurospora crassa*, and *Saccharomyces cerevisiae* [(Barnett et al., 1988; Toth and Coffino, 1999) for review see (Ivanov et al., 2006)]. The regulator of Odc is the protein ornithine decarboxylase antizyme (Az) (Hayashi et al., 1996). Az interacts with Odc to be degraded by the proteasome in an ubiquitin independent manner (Zhang et al., 2003). Among fungi, the regulatory mechanism of Az is conserved [for review, see (Sorais et al., 2003)]. As indicated above, it was demonstrated that in *S. cerevisiae*, the degradation of Odc occurs without ubiquitination, as also happens in mammalian cells (Gandre and Kahana, 2002; Ivanov et al., 2006), and the identification of genes encoding Az from other fungi, suggests that the mechanism may be widely distributed in these organisms (Ivanov et al., 2006; Ivanov and Atkins, 2007). A peculiarity of fungi is that they present a single antizyme ortholog, while mammalian cells posses several antizyme encoding genes (Coffino, 2001a,b; Kurian et al., 2011). As expected, it is known that Az is positively regulated by polyamine levels. The mechanism for this regulation involves a translational frameshift in the open reading frame (ORF) in the encoding gene, through which a second ORF that encodes the active protein is established. In *S. cerevisiae* an unusual mechanism for the control of Az synthesis was unveiled (Kurian et al., 2011). Accordingly, the authors made the surprising discovery that at low polyamine levels Az acquires a conformation that arrest its own synthesis, but high concentrations of polyamines bind, not to the regulatory region of the gene, but directly to the Az polypeptide, thus avoiding that it acquires such conformation, and promoting the completion of its synthesis Other regulatory mechanisms of the metabolism of polyamines occur at the levels of Odc, Ssat, and Pao, due to their early and rapid responses to external stimuli (Vujcic et al., 2003; Wallace et al., 2003). Additionally, the existence of regulatory sequence elements in the 5′ and 3′ of *ODC* regions in *N. crassa* are related with changes in the rate of synthesis of Odc, and with changes in the abundance of *ODC* mRNA (Williams et al., 1992; Hoyt et al., 2000).

Contrary to fungi, plants posses several copies of the genes involved in polyamine metabolism which increase the complexity of polyamine regulation. As an example, in *Arabidopsis thaliana* there are two genes encoding *Adc1* and *Adc2*, the first one is presented in all tissues and it is constitutively expressed, while the second one responds to some abiotic stresses (Soyka and Heyer, 1999; Perez-Amador et al., 2002). Likewise, *Spd1* and *Spd2* are the genes encoding spermidine synthase in *A. thaliana* (Imai et al., 2004; Ge et al., 2006).

Regard SAMDC, it is synthesized as a proenzyme, it has been demonstrated that putrescine induces the cleavage of the proenzyme in a specific amino acid to produce the active and mature enzyme (Pegg et al., 1998). In plants there is an additional regulation control at transcriptional level. Plant *Samdc* contain a tiny 5′-uORF and introns in 5′leader sequence that regulate their expression (Hu et al., 2005). The absence of Az homolog in plant genomes corroborates the predominance of SAMDC as the regulator of polyamine homeostasis (Illingworth and Michael, 2012).

### Interactions with other metabolic pathways

The pleiotropic effects observed in polyamine mutants may be due to the relationships existing between polyamines and other metabolic pathways. As an example, ornithine, the precursor of putrescine not only is considered a key regulator of polyamine biosynthesis, but it may also regulate the pathways for glutamate transformation to arginine and to proline. Indirectly, it can also regulate putrescine catabolism, contributing to the aminobutiric acid content of the cells, since putrescine can be converted into Δ^1^-pyrroline by an amino oxidase, and this compound is metabolized to γ-aminobuyrate by pyrroline dehydrogenase (Seiler et al., 1979; Fogel et al., 1981; Majumdar et al., 2013). Another compound related to polyamines is *S*-adenosylmethionine (SAM), which is essential for the synthesis of polyamines. *S*-adenosylmethionine synthetase (Sams; EC 2.5.1.6) catalyzes the biosynthesis of SAM from ATP and L-methionine (Tabor and Tabor, 1984). In *S. cerevisiae*, methionine regulates the expression of the two *SAMS* genes (*SAMS1* and *SAMS2*) (Thomas et al., 1988); and in turn *SAMS2* gene is subjected to the inositol-choline regulation given by the octameric sequence 5′-CATRTGAA-3′ contained in its upstream promoter region (Kodaki et al., 2003). Genes encoding Sams are evolutionarily well conserved (Mautino et al., 1996). In *S. pombe* mutants, the absence of Sams affected cell growth, mating and sporulation, and by over-expressing the gene, growth of the cells became methionine-sensitive (Hilti et al., 2000).

5′-Methylthioadenosine (MTA), is a product of SAM catabolism during polyamine biosynthesis (Heby, 1981). More than 98% of MTA in *S. cerevisiae* is a by product of SAM, originated from the polyamine biosynthetic pathway (Avila et al., 2004). MTA can affect gene expression, proliferation, differentiation and apoptosis, and these effects may be due to the inhibitory effect that intracellular accumulation of this nucleoside has over polyamine biosynthesis *in vivo* (Raina et al., 1982; Garcea et al., 1987); in addition in *S. cerevisiae*, MTA causes a specific inhibition of spermidine synthase (Chattopadhyay et al., 2006a).

Regard plants, SAM is the precursor of ethylene, hence polyamines, DNA methylation and ethylene share it as a common precursor (Pandey et al., 2000). Polyamines are precursors of many plant secondary metabolites such as nicotine and tropane alkaloids (Martin-Tanguy, 2001). Furthermore, plant polyamine metabolism is also connected with the production of nitric oxide and GABA [Reviewed by Alcazar et al. (2010)]. The regulation of polyamine biosynthesis, proline and cytokinins by abscisic acid as well as ethylene during UV-B stress and salt stress has been documented (Rakitin et al., 2009; Xue et al., 2009; Shevyakova et al., 2013).

## Polyamines and stress

### Tolerance of fungal mutants affected in polyamine metabolism to different stress conditions

The study of the stress response in fungal mutants affected in polyamine metabolism was started with *S. cerevisiae*, where the authors had in mind evidences indicating some roles of polyamines in the protection of the cell and cell components, and cell differentiation (Balasundaram et al., 1993; Chattopadhyay et al., 2006b; Watanabe et al., 2012). In general the ability to deal with stress is diminished in mutants affected in any of the genes encoding enzymes related with polyamine metabolism. *U. maydis* is a well studied system in this regard, proving to be an important model organism for understanding polyamine metabolism, especially since this phytopathogenic fungus lacks spermine (Valdes-Santiago et al., 2009).

In general, fungal spores are rather more resistant to different environmental stresses than vegetative cells. However, in the early steps of germination, when they loose their unique spore wall, they become more sensitive to different environmental stresses (Herman and Rine, 1997; Joseph-Strauss et al., 2007). Interestingly, it has been described that polyamines affect positively spore germination in *Glomus mosseae, Rhizopus stolonifer, Botryodiplodia theobromae, Gigaspora rosea*, and *Glomus etunicatum* (Nickerson et al., 1977; El Ghachtouli et al., 1996; Sannazzaro et al., 2004; Cheng et al., 2012). Inhibition of polyamine metabolism gave rise to an inhibition of spore germination and germ tube growth in fungi such as *Uromyces phaseoli* (Galston, 1989). Genes related with polyamine transport have been implicated in the germination process, and it was described that these genes are up-regulated compared to the vegetative state (Ruiz-Herrera, 1994; Dembek et al., 2013). These results suggest a relationship between polyamines and changes in the expression of genes related to stress. During a study of *S. cerevisiae* sporulation-specific genes, two divergently transcribed genes *DIT1* and *DIT2*, were found to be repressed during vegetative growth via a common negative regulatory element, referred to as NER^DIT^ (Friesen et al., 1997). The authors reported that the spermidine synthase gene was required for complete repression through NER^DIT^, and cells that could not synthesize spermidine not only failed to support complete repression, but also had modest defects in repression of other genes, suggesting that spermidine could modulate gene expression (Friesen et al., 1998). However, there is not yet a report about gene expression response of fungi to different environmental stresses that reveal information on what is the mechanism through which polyamine are operating in this context.

### Role of physiological polyamines on osmotic stress

*In silico* phylogenetic analyses have revealed that central components of the osmotic, oxidative and cell wall stress signaling pathways are relatively well conserved in fungi (Bahn et al., 2007; Nikolaou et al., 2009). Nevertheless, knowing that polyamines are important in the response to stress, and that their metabolic pathway has been conserved among all living organism; we may suggest that a principal role of polyamines is to promote the restoration of cellular homeostasis allowing survival under stressful conditions. In *S. cerevisiae*, the expression of the major permease for high affinity polyamine import coincided with the osmotic stress imposed by high concentration of NaCl, KCl, or sorbitol (Lee et al., 2002; Aouida et al., 2005). Also in yeast, it was observed that the serine/threonine protein kinases Ptk1p and Ptk2p were involved in the regulation of spermine uptake, and that disruption of *PTK2* gave rise to salt tolerance, while Ptk2p or Sky1p (another serine/threonine kinase that was found in spermine tolerant strains) over-expression, led to increased salt sensitivity (Erez and Kahana, 2001). These and other data clearly reveal that polyamines have a role in osmotic stress response, probably by regulation of the expression of osmotic stress signaling via protein kinases (Auvinen et al., 1992; Shore et al., 1997; Flamigni et al., 1999).

As would be expected, it is known that the lipid composition of the membrane affects the osmotic stability of the yeast plasma membrane (Allakhverdiev et al., 1999). In consequence, it was important to establish if the sensitivity of polyamine mutants to different kinds of stress was due to a defect at the membrane level or to an indirect alteration of other signaling pathways controlled by polyamines. In this sense, data exist showing that osmotic shock in different systems affects the intracellular levels of polyamines. For example, it was reported that under osmotic shock the content of putrescine was increased up to 60-fold in oat, barley, corn, wheat, and wild oat leaves (Flores and Galston, 1982); and the induction of Odc activity under hypo-osmotic stress leading to an increase in the polyamine pool was observed in L1210 mouse leukemia cells (Poulin et al., 1991). The effect of a high osmotic concentration was tested in *U. maydis* mutants affected in the gene encoding spermidine synthase (*spd*), and in double mutants affected also in the ornithine decarboxylase gene (*odc/spd*). Both simple and double mutants showed no significant differences in their growth rate in the absence of salt stress, when compared to the wild type, but growth rate of the same cells treated with 1 M KCl or 0.3 mM SDS was severely inhibited in comparison with the wild type strain. This result demonstrates a role of spermidine in protecting the fungus from different deleterious factors that affect the cell membrane (Valdes-Santiago et al., 2009). When mutants affected in the polyamine oxidase gene (*pao*), and double mutants affected in ornithine decarboxylase and polyamine oxidase (*odc/pao*) were subjected to ionic or osmotic stress (1 M NaCl, 10 mM LiCl, or 1 M sorbitol), only the double mutant displayed a sensitive phenotype, whereas the simple *pao* mutant showed no differences to the wild type strain (Valdes-Santiago et al., 2010).

To understand the effects of polyamine deficiency on the phenotypic alterations suffered by the cell subjected to osmotic stress, it is important to take into consideration the existence of specific mechanisms involved in cation handling, and the possible effect of some pathways that interact with polyamine metabolism. This fact was clearly demonstrated by the comparison of the behavior of *U. maydis S*-adenosylmethionine decarboxylase (*samdc*) and *spd* mutants under stress conditions. Growth rate of both mutants was substantially inhibited by 1 M NaCl, but only the *samdc* mutant was affected by 10 mM LiCl. A fundamental difference between *samdc* and *spd* mutant is that, unlike the wild-type strain, they accumulate high levels of SAM, but *spd* mutants also accumulate SAM. The other metabolite involved in the pathway is dcSAM, which was 46-fold increased in the *spd* mutants (Valdés-Santiago et al., 2012b). The authors explained these differences in the phenotype in relation to their different levels of SAM and dcSAM, which could affect DNA methylation, and other different cellular functions, plus the possibility that sodium and lithium are managed by different transporters.

**Figure 2:**
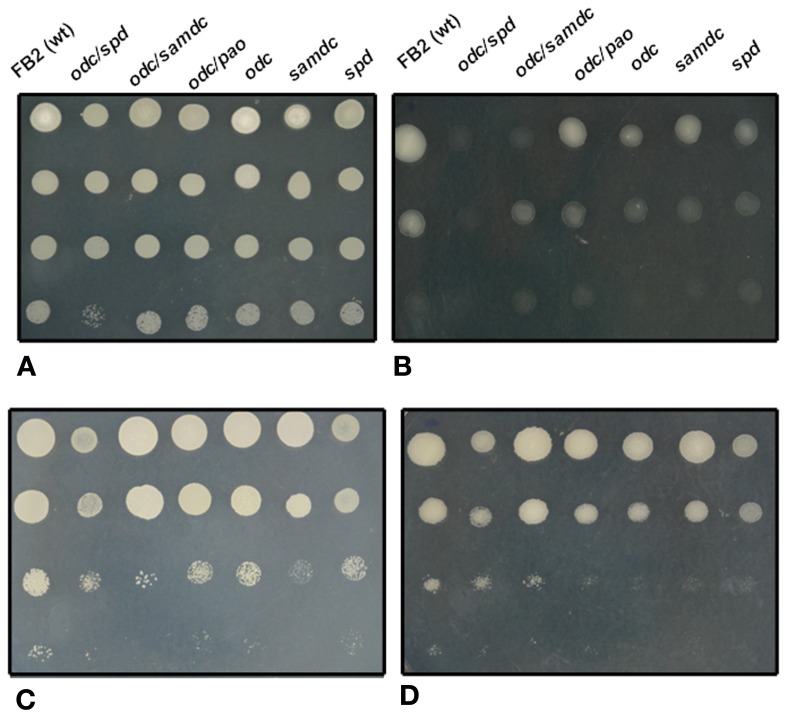
**Comparative analysis of different stress conditions on the growth of wild type, and simple and double mutants of *U. maydis* affected in different steps of polyamine metabolism**. After depletion of polyamine pools, cell suspensions were decimally diluted and inoculated on solid minimal medium containing 20 mM lysine and 0.1 mM spermidine. After 48 h of incubation at 28°C (except in C), photographs were taken. **(A)**, control; **(B)**, 1.5 M KCl added to the medium; **(C)**, as **(A)**, but incubated at 37°C; **(D)**, 1 M NaCl added to the medium.

A further example of the specificity of polyamines in osmotic stress was the observation that in *Synechocystis* sp., a cyanobacterium, salt stress induced an increase in the spermine content, whereas an osmotic stress induced a moderate increase in the total spermidine content (Jantaro et al., 2003). Interestingly, this response was correlated with an increase of arginine decarboxylase mRNA levels, and an increase in the uptake of putrescine and spermidine (Incharoensakdi et al., 2010). In this sense, we may cite that simple or double *U. maydis* polyamine mutants presented a variety of stress sensitivities to osmotic stress (Figures 2B,D), compared with wild type and same mutants without stressful agent (Figure 2A).

In plants it has been well established that general polyamine biosynthesis is modified under salt stress at transcriptional level [for review see (Mutlu and Bozcuk, 2007; Liu et al., 2008; Alcazar et al., 2010; Gupta et al., 2013)]. However, spermine seems to have a specific role considering that, this is the polyamine reported to be more affected regardless of species, varieties and plant tissue studied. To mention some examples, plants such as *A. thaliana* unable to produce spermine showed hypersensitivity to high levels of NaCl and KCl and the mechanism behind polyamines protectection of salt stress has been studied. Some reports suggest that, polyamines improve ionic equilibrium by modifying the plasma membrane to overcome the osmotic stress since the expression of some genes belonging to signaling pathway related with salt stress were not altered in spermine deficient mutant. Moreover, spermine seemed to modulate Ca^2+^ permeable channels and change Ca^2+^ allocation restraining the entry of Na^+^/K^+^ to the cytoplasm (Yamaguchi et al., 2006; Janicka-Russak et al., 2010; Najmeh et al., 2012; Velarde-Buendia et al., 2012). Other reports described that spermidine or putrescine are the polyamines conferring protection under salt stress, although it is difficult to support this conclusion, since putrescine or spermidine can be inter-converted (Quinet et al., 2010; Saleethong et al., 2011). In this respect, mutants are very effective in avoid confused results since they can define whether polyamines are produced by *de novo* synthesis or back-conversion (Valdes-Santiago et al., 2010).

### Oxidative stress response

Fungi, as well as other microorganisms, must deal with the danger of oxidative stress under different scenarios. An important example is the oxidative killing of fungal cells by the host defense mechanisms, and sometimes the ability to proliferate in the host has been correlated with the expression of redox active enzymes such as catalase (Wysong et al., 1998; Moye-Rowley, 2003). Accordingly, it has been concluded that one of the polyamine functions is the cell protection from damage caused by ROS (Rider et al., 2007; Cerrada-Gimenez et al., 2011). In the case of *E. coli*, oxidative stress induces the expression of catalase, hydroperoxide reductase and glutathione reductase (Storz and Imlay, 1999; Chattopadhyay et al., 2003a), and interestingly, putrescine is up-regulated the bacterium in a concentration-dependent manner with the expression level of the transcription factor controlling the genes already mentioned (Kim and Oh, 2000; Tkachenko et al., 2001). In the case of *U. maydis* it was observed that *pao* and *odc* polyamine mutants grown on agar plates where 0.8 mM H_2_O_2_-containg paper disks were placed, showed halos of inhibition wider in comparison with the wild-type strain; i.e., they were more sensitive than the wild type to the oxidative stress (Valdes-Santiago et al., 2010).

In fungi in general the mechanism regulating their response to oxidative stress has been described to involve the control of oxidant-responsive factors, such as Yap1p at level of cell localization and by regulation of enzyme activity via protein phosphorylation (Moye-Rowley, 2003). When *S. cerevisiae* was affected in the production of spermidine and spermine because of a deletion of the *Samdc* gene; there occurred a loss in cell viability when cultures were incubated under an oxygen atmosphere (Balasundaram et al., 1993).

It has also been shown that yeasts affected in the production of spermidine accumulate ROS, and show an increased sensitive to oxidative damage. In this regard, polyamine-deficiency in *S. cerevisiae* induced accumulation of ROS that led to the development of an apoptotic phenotype (Chattopadhyay et al., 2006b). These authors showed that one of the polyamine functions was the protection to the accumulation of ROS. There is yet to know whether polyamines act by affecting the enzymes involved in the synthesis or degradation of ROS, or by a direct interaction with ROS. Some evidences point out to some hypothetical polyamine-binding sites in proteins involved in these different processes (Watanabe et al., 2012). In this same respect, it has been proposed that spermine acts by direct scavenging of reactive agents (Ha et al., 1998; Fujisawa and Kadoma, 2005), whereas other authors have suggested an inhibition of the activity of NADPH oxidase (Papadakis and Roubelakis-Angelakis, 2005), and a role of polyamines as regulators of the MAPK signaling pathway (Stark et al., 2011).

In plants, a swift accumulation of ROS is presented under stressful conditions (Gill and Tuteja, 2010b; Suzuki et al., 2012); and it has been observed that intracellular ROS produced by polyamine catabolism are essential during development. Thus, it has been described that H_2_O_2_ generated during their oxidation and back-conversion serve as a signaling molecule correlated with plant defense and stress response (Moschou and Roubelakis-Angelakis, 2011; Pottosin et al., 2012). Accordingly, in *Salvinia natans* response to salinity there occurs an interaction between ROS formation, and the expression of *Adc*, *Samdc*, *Spd*, and *Spm* as well as *Pao* (Tanou et al., 2013).

### Temperature stress

Temperature has critical effects on microbial metabolism and cellular composition (Bennett et al., 1992; Feder and Hofmann, 1999; Fargues and Luz, 2000; Gavito and Azcon-Aguilar, 2012). It has been suggested that polyamines are involved in the stabilization of cellular components at high temperatures; for example a *Tapesia yallundae odc* null mutant exhibited temperature-dependent growth: at high temperatures, hyphal elongation was more restricted in comparison to the wild type, and the hyphae were thinner, less melanized, and grew sparsely (Mueller et al., 2001). Similarly, an *S. cerevisiae* mutant unable to synthesize spermidine or spermine, because it was affected in the gene encoding *S*-adenosylmethionine decarboxylase, was more sensitive to elevated temperatures than the parental strain (Balasundaram et al., 1996). The opposite takes place when polyamines are accumulated. Under these conditions, the cells present resistance to high temperature stress (Cheng et al., 2009). Simple and double *U. maydis* mutants affected in polyamine metabolism also presented a temperature sensitive phenotype in agreement with the reports above mentioned (see Figure 2C).

In plants, polyamine alterations have been correlated with temperature changes; under cold treatment putrescine levels were increased together with the expression of *Adc1*, *Adc2*, and *Samdc2* in *A. thaliana* (Urano et al., 2003; Cuevas et al., 2008), while the addition of putrescine had a positive effect over cotton seed subjected to high temperature (Bibi et al., 2010). These results are in agreement with reports on *Arabidopsis thaliana*, where a mutant affected in the gene encoding spermine synthase was hypersensitive to heat shock; whereas an overexpression of spermine synthase-encoding gene conferred thermotolerance to the cell (Sagor et al., 2013). In the same study it was established a direct correlation between spermine content and the expression of heat shock genes and proteins suggesting that there is a control of polyamines at transcriptional and translational levels to induce the protection of plants under temperature stress. It is interesting to notice that although *U. maydis* does not contain spermine, spermidine covers the function to resist temperature stress (Figure 2C).

In the same line, it has been suggested that he mechanism behind the sensitivity of polyamine-deficient cells could be related to the stability that polyamines may provide to some cell components (Schuber, 1989).

### Polyamines in stress produced by pathogen-host interactions

It is well known that during the course of host colonization, fungal pathogens need to overcome a wide range of challenges such as oxidative burst, which results in the production and accumulation of ROS. It has been proposed possible roles for polyamines and polyamine catabolism in plant resistance to pathogen infection (Walters, 2003). In this context, plants would activate polyamine oxidation to produce hydrogen peroxide, which would lead plant defense mechanisms. Yoda et al. (2003) confirmed these data, when they correlated hypersensitive response with the accumulation of polyamines in the apoplast of *Arabidopsis thaliana* infected with *Pseudomonas syringae*, and of rice infected with *Magnaporthe grisea* (Yoda et al., 2003). Expression of *Odc*, *Adc*, and *Samdc* genes in *Theobroma cacao* were induced under different stresses such as, drought and infection with *Phytophthora megakarya*, or the necrosis inducing protein Nep1 from *Fusarium* oxysporum while *Spds* and *Sps* were not changed (Bae et al., 2008). In the same manner, H_2_O_2_ produced by Paos during hypersensitive response provoke disease tolerance against *Pseudomonas syringae pv* tabaci and *Phytophthora parasitica* var nicotianae (Yoda et al., 2003; Moschou et al., 2009).

Spermine has been proposed as the polyamine that contribute to defense signaling against plant pathogens through the regulation of defense-related genes. In this sense, an *A. thaliana* mutant that overexpressed *Sps* gene, displayed higher resistance to infection with *Pseudomonas viridiflava*, whereas a mutant with low spermine levels showed hyper sensitivity; interestingly the over-expression of *Sps* was accompanied by up-regulation of genes involved in disease resistance protein, as well as several transcription factors (Cona et al., 2006; Kusano et al., 2008; Gonzalez et al., 2011). Likewise, spermine-responsive genes were detected in *A. thaliana* during infection with cucumber mosaic virus, and interestingly, blocking of spermine oxidation abolished induction of these genes (Mitsuya et al., 2009). In general, response from pathogenic organism with distinct strategies to cause disease either necrotrophic or biotrophic, is essentially analogous, as was demonstrated with *Sclerotinia sclerotiorium* and compared with some *Pseudomonas species* or *U. maydis* (Marina et al., 2008; Rodriguez-Kessler et al., 2008; Rodriguez-Kessler and Jimenez-Bremont, 2009). In these two cases what was observed was the induction of polyamines connected with the activation of genes encoding polyamine biosynthesis enzymes. Nevertheless, it is important to mention that changes in plant polyamine metabolism by transgenic strategies allow the modification of plant responses to pathogenic organisms. The expression of yeast *Spe* gene and accumulation of polyamine levels in tomato led to an increasing of sensitivity to the attack by *Botrytis cinerea*, but not by *Alternatia solani* and the normal response to the attack was restored by polyamine inhibitors (Marina et al., 2008; Nambeesan et al., 2012).

From the side of the pathogen, the state of polyamine metabolism affects their interaction with the host. When this *Streptococcus pneumoniae* was affected in polyamine transport, the mutant strain showed attenuation in virulence in a murine model. And it has been reported that human bacterial pathogens use different strategies related with polyamines to infect their hosts (Ware et al., 2006; Di Martino et al., 2013). A polyamine mutant of *Salmonella enterica* serovar *typhimurium*, was unable to invade and survive intracellularly in *Caenorhabditis elegans*, and showed no systemic infection in a mouse model of typhoid fever (Jelsbak et al., 2012). *Francisella tularensis, Yersinia pestis* and *Vibrio cholerae* are other systems in which synthesis and transport of polyamines were found to be important for virulence (Wortham et al., 2010; Russo et al., 2011; Goforth et al., 2013). Regarding fungi, a *spe* mutant of the thermally dimorphic fungus *Penicillium marneffei*, a pathogen of immune compromised persons, showed defects in pathogenesis, conidiogenesis, spore germination, and growth. These results led to suggest that the spermidine biosynthetic may serve as a potential target for combating infections (Kummasook et al., 2013). Agreeing with these ideas, in another pathosytem reviewed by Valdés-Santiago et al. (2012b): *U.maydis*-maize, it was observed that *samdc* mutants of the fungus were avirulent.

## General conclusions and future perspectives

Along this article, evidences indicate multiple roles of polyamines in cell survival during stress. A strategy toward the knowing of the mechanism behind polyamines action should include firstly the clear identification of the specific roles of each polyamine i. e., in *U. maydis* putrescine might be controlling stress response since the mutant (*odc/pao*) was over sensitive to stress, in comparison to the wild type and and *pao* mutant. Also, spermidine appeared to control differentiation of this fungus, since mutant it was able to carry out a dimorphic transition only when supplied with high concentration of spermidine (Valdes-Santiago et al., 2010). Once the specific roles of each polyamine are known, it will be possible to distinguish the role of other polyamines in the general aspect of cell physiology. This review clearly stresses the fact that changes in polyamine metabolism affect the response of fungi to different types of stress. This fact by itself is an evidence of the importance of polyamines in cell survival.

Although a large advance in our understanding on polyamine metabolism in fungi has taken place in recent years, some aspects are still poorly understood, especially regarding polyamine transport, distribution in the cell, and regulation of metabolism. It is also clear that the role of polyamines in stress-response mechanisms in fungi, and their mode of action have been insufficiently analyzed. We consider that it is necessary to have a global view of the physiology of stress response in fungi that includes polyamines as an important player, possibly using mutants affected in different steps of polyamine metabolism and its regulation, and studies of the relationships of polyamine metabolism with other important metabolic and regulatory processes of the cell. If this expectancy is fulfilled, undoubtedly that fungi will become important models that could help to unravel the mechanism of the protection exerted by polyamines to stress in general.

### Conflict of interest statement

The authors declare that the research was conducted in the absence of any commercial or financial relationships that could be construed as a potential conflict of interest.
